# Encapsulation of palladium porphyrin photosensitizer in layered metal oxide nanoparticles for photodynamic therapy against skin melanoma

**DOI:** 10.1088/1468-6996/16/5/054205

**Published:** 2015-10-09

**Authors:** Zih-An Chen, Yaswanth Kuthati, Ranjith Kumar Kankala, Yu-Chuan Chang, Chen-Lun Liu, Ching-Feng Weng, Chung-Yuan Mou, Chia-Hung Lee

**Affiliations:** 1 Department of Life Science and Institute of Biotechnology, National Dong Hwa University, Hualien, 974, Taiwan; 2 Department of Chemistry, National Taiwan University, Taipei 10617, Taiwan

**Keywords:** layered double hydroxide, nanoparticles, photodynamic therapy

## Abstract

We designed a biodegradable nanocarrier of layered double hydroxide (LDH) for photodynamic therapy (PDT) based on the intercalation of a palladium porphyrin photosensitizer (PdTCPP) in the gallery of LDH for melanoma theragnosis. Physical and chemical characterizations have demonstrated the photosensitizer was stable in the layered structures. In addition, the synthesized nanocomposites rendered extremely efficacious therapy in the B16F10 melanoma cell line by improving the solubility of the hydrophobic PdTCPP photosensitizer. The detection of singlet oxygen generation under irradiation at the excitation wavelength of a 532 nm laser was indeed impressive. Furthermore, the *in vivo* results using a tumour xenograft model in mice indicated the apparent absence of body weight loss and relative organ weight variation to the liver and kidney demonstrated that the nanocomposites were biosafe with a significant reduction in tumour volume for the anti-cancer efficacy of PDT. This drug delivery system using the nanoparticle–photosensitizer hybrid has great potential in melanoma theragnosis.

## 1. Introduction

Photodynamic therapy (PDT) has great potential in cancer therapy as an alternative to surgery. During PDT, the singlet oxygen generated has a very short lifespan (<3.5 microseconds) with limited diffusion rate, hence the damage from the resulting photo-oxidation is confined only to the site of photosensitizer accumulation [1]. Despite its unusual success rate, there are certain limitations for photosensitizers such as hydrophobicity, which leads to aggregation in a biological buffer, thereby hindering their parenteral administration [2] and thermal degradation upon light exposure *in vivo* [3]. The incorporation of photosensitizers into a nanocarrier has been proposed most often to address the stability and biocompatibility issues [4]. These encapsulated nanohybrids have been shown to increase the phototoxicity compared to the free photosensitizer [5–7], due to high cellular uptake and increased accumulation in the tumour [8]. Additionally, the higher surface area-to-volume ratio of the tiny nanocontainers increases the surface area exposed to the surrounding medium, which aids in greater photosensitizer release rates [9].

In recent years, inorganic nanomaterials have increased applications in drug delivery as well as in intracellular bioimaging [10–14]. A few stability limitations associated with polymer- or liposome-based systems [15] have motivated researchers to focus on inorganic nanocarriers. These inorganic nanocontainers are highly stable with great biocompatibility, well-ordered size, shape and porosity [16]. In addition, these materials can be easily functionalized for selective targeting, which would allow the selective accretion of photosensitizers at the site of action rather than non-target tissues thereby decreasing the toxic side effects. Yang *et al* have used aggregated gold nanoparticles intracellularly for surface plasmon resonance-enhanced singlet oxygen generation in PDT [17]. Cao *et al* have developed a novel targeted cancer PDT through the uptake of photosensitizer-loaded hollow silica nanoparticles carried by mesenchymal stem cells (MSCs) [18]. Graphene oxide has also been used for successful PDT [19]. Among the inorganic nanomaterials studied, layered double hydroxide nanoparticles (LDHs) have been one of the more fascinating developments in biomedical applications. Their unique structure consisting of positively charged layers has allowed applications to deliver a wide variety of biomolecules [20–24]. Furthermore, their controllable anion exchange property with pH response has aided in the design and development of an intelligent cargo carrier system that can transport negatively charged drugs [25] to the targeted sites on demand with control over the rate of delivery [26]. The inter-layered structures of LDHs help the incorporation of not only several types of drug molecules [27] but also the simultaneous loading of imaging agents along with the drugs [28] for combined chemotherapy and bio-imaging. The fabrication of LDH materials has been exploited to gain the benefits in a nano construct with multiple functions. This combination has shown to impart additional benefits such as targeting ability [29], controlled drug release [30], easy particle functionalization [31], improved biocompatibility [32] and enhanced photostability and contrast efficacy of imaging agents [33]. These properties make LDHs attractive for PDT applications and permit high loading of photosensitizers while the avid cellular uptake enables substantial intracellular accretion of photosensitizers for ideal PDT [34].

LDHs are a class of layered materials with positively charged layers and exchangeable charge-balancing anions in the interlayer region. LDHs are commonly represented by the formula [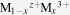 (OH)_2_]^*q*+^(X^*n*−^)_*q*/*n*_·*y*H_2_O, and they can be synthesized and assembled to form various nanoscale structures. Ariga *et al* summarized the concepts of layer-by-layer (LbL) nanoarchitectonics by self-assembly from bottom-up approaches. The use of the LbL deposition process to produce multilayer films with nanoscale structures can be applied in many fields such as materials science, chemistry, physics and biomedicine [13]. Further, Xu *et al* summarized the theoretical and experimental concepts for exploring the internal quantum degrees of freedom electrons from two-dimensional metal-layered materials. These unique physical properties of electron spin can also be applied to new electronics [14]. LbL applications in LDHs were extended by Gunjakar *et al* using opposite charges of Zn–Cr-LDH and layered titanium oxide 2D nanosheets assembled into mesoporous heterolayered nanohybrids, which showed a strong absorption to visible light for the improvement of photocatalytic activity [35]. Thin films with multicolour emission were synthesized using an LbL assembly of two different wavelengths of CdTe quantum dots with LDH. These thin films were stacked as a periodic ordered structure, which can further enhance energy transfer effects and can be applied in the fields of biological sensors and devices [36]. Further, the loading amounts of anions into the interlayer gallery of LDHs were increased by Li *et al* to raise the anionic concentration and hydrothermal temperature which can generate a greater basal spacing. Thus, unilamellar nanosheets were produced by delaminating layered rare-earth hydroxide-dodecylsulfate (LRH-DS) in formamide. Further, spin-coating for self-assembly of the unilamellar nanosheets and annealing can generate an oriented oxide film with a strong photoluminescence property [34]. In addition, Mg_2_Al-LDH with sodium dodecyl sulfonate (SDS) as the counter-ion molecule has been used for incorporation of zinc phthalocyanines (ZnPc) into the LDH resulting in a highly efficient PDT effect both *in vitro* and *in vivo* [37]. The hydrophilic sensitizer ZnPc linked with sulfonatophenoxy groups has also been exchanged/intercalated in LDHs. This organic–inorganic nanohybrid can release photosensitizers at a more acidic pH of tumour tissues, and furthermore the PDT activity was augmented through an efficient cell-uptake mechanism [38]. Stefanakis *et al* addressed the synthesis of amine-modified Gd_2_(OH)_5_NO_3_ nanosheets to conjugate with a photosensitizer (rose bengal), which can improve proton relaxivity and photooxidative activity and shows promising applications in PDT and as a magnetic resonance imaging (MRI) contrast agent [39]. The PDT approach can trigger the reactive oxygen species (ROS) to exterminate the cancer cell, which motivated Merchan *et al* to immobilize a photosensitizer in Zn_2_Al-LDH to increase the photostability and photobactericidal properties [40]. Very recently, Wang *et al* developed a combination of Pt(IV) prodrugs and photosensitizers intercalated in the LDH to enhance the cytotoxic effects in cisplatin-resistant cancer cells [41].

Palladium porphyrin, a synthetic metalloporphyrin has been extensively studied for *in vivo* quantitative oxygen sensing and imaging [42]. The PdTCPP triplet state is highly persistent and responsible for singlet oxygen generation at good quantum yield. This is directly proportional to the accessibility of phosphorescence quenching of the excited PdTCPP by oxygen. It is possible to switch its function from phosphorescence probe to photosensitizer (PS) by increasing the irradiation energy. PdTCPP is protected from decomposition by its incorporation into LDHs to prevent the undesirable release of the photosensitizer in the targeted site. PDT is efficient only when the short-lived singlet oxygen species generation and action are executed within the cell. The positively charged LDH-carrying photosensitizer could be highly endocytosed, interacting with negatively charged biological membranes for treating melanoma [43]. The layered matrix can offer a shielding effect to the photosensitizer which can limit the PS degradation and entry into the systemic circulation. The high surface area and the positive charge of the LDH interlayer matrix would permit the delivery of PS in high concentrations, thereby improving the efficacy of PdTCPP. In addition, the biodegradable framework of LDHs suggests better biocompatibility than other Pd-based porphyrin systems [5] in nanooncology [30].

**Figure 1.**
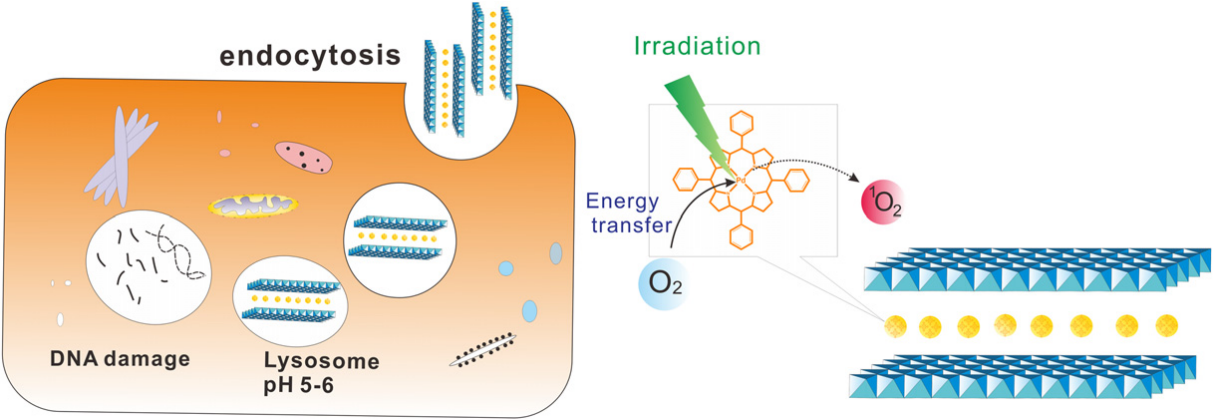
Schematic representation of the uptake of LDH–PdTCPP nanocomposites by cell and resultant apoptosis by photodynamic therapy.

In this work, palladium porphyrin (Pd-meso-tetra(4-carboxyphenyl)porphyrin (PdTCPP) intercalated LDH nanohybrids (figure 1) were synthesized for PDT, which could exhibit the following advantages: (i) homogeneous distribution of the photosensitizer (PS) at the molecular level, thereby increasing the quantum yield; (ii) imparting hydrophilicity and enhancing the biocompatibility of the PS molecules that favour cellular uptake; and (iii) enhancing chemical and photostability of the PS molecules. We summarize the synthesis of the nanohybrids and evaluate their *in vivo* PDT efficacy in mouse skin melanoma (B16F10 cancer cells).

## 2. Materials and methods

### 2.1. Materials

All solvents and chemicals obtained were used without further purification. Anhydrous aluminium chloride (AlCl_3_), magnesium chloride (MgCl_2_.6H_2_O), Pd-meso-tetra(4-carboxyphenyl) porphyrin (PdTCPP), fluorescein isothiocyanate (FITC), formaldehyde (HCHO), KBr (FT-IR grade), diamidino-2-phenylindole dihydrochloride (DAPI) and an *in vitro* lactate dehydrogenase kit were obtained from Sigma-Aldrich. 3-aminopropyl-trimethoxysilane (APTS), sodium hydroxide (NaOH), and dimethyl sulfoxide (DMSO) were purchased from Acros Organics Ltd Dulbecco’s modified Eagle’s medium (DMEM) and foetal bovine serum (FBS) were obtained from GIBCO/BRL Life Technologies (Grand Island, NY, USA). The comet assay kit was purchased from Trevigen manufacturers (Gaithersburg, MD, USA).

### 2.2. Characterization

Fourier transform infrared (FT-IR) spectra were recorded on a Bruker Alpha spectrometer with a dried KBr pellet. Particle size distribution as well as zeta (*ζ*)-potential was measured by Malvern Nano-HT Zetasizer. Powder x-ray diffraction (PXRD) analysis of the samples was carried out using an XRD D8 advanced diffractometer (Bruker). Thermogravimetric analysis (TGA) and differential thermal analysis (DTA) curves were recorded on universal V4.5A TA Instruments. Transmission electron microscope (TEM) images were taken on a Hitachi H-7100 instrument. Nitrogen adsorption–desorption isotherms were plotted for surface area and pore size distribution using the surface area analyzer on a Micrometric ASAP 2020 at the temperature (−196 °C).

### 2.3. Instruments

UV–vis absorbance was recorded on Genequant-1300 series spectrophotometer. MTT absorbance was recorded using Perkin Elmer’s EnSpire Multi-label Plate Reader (Santa Clara California, USA). Centrifugation during nanomaterials synthesis was performed at an appropriate temperature using Hermle Z 36 HK (Wehingen, Germany) instruments.

### 2.4. Synthesis of LDHs

LDHs were prepared by co-precipitation following hydrothermal treatment [44, 45]. In brief, the pristine LDH was prepared by adding 10 mL of mixed salt solution containing MgCl_2_.6H_2_O (3.0 mmol) and AlCl_3_ (1.0 mmol) quickly into 40 mL of NaOH solution (0.15 M) to co-precipitate under vigorous stirring for 10 min under nitrogen purge. Solutions were prepared using decarbonated water under high precautions to prevent the contamination. The resultant slurry (LDH) was obtained by spinning down, followed by washing twice with deionized water. The slurry was then redispersed in deionized water (40 mL) and transferred into a hydrothermal flask with Teflon lining and maintained at 100 °C for 16 h to give a stable homogeneous LDH suspension after air-cooling. The resultant solid was separated by centrifugation, washed twice with water and ethanol, and stored in ethanol (99.5%) to prevent microbial contamination.

### 2.5. Synthesis of LDH–PdTCPP

PdTCPP was loaded into the LDH using both the ion-exchange method and the following procedure. Pd-meso-tetra (4-carboxyphenyl) porphyrin (40 mg, (0.044 mmol)) was dissolved in methanol (10 mL) and the pH was adjusted to 8.0–8.5. Then, 10 mL of the resulting reaction mixture was added to 20 mL of the LDH solution (10 mg mL^−1^ in methanol) and stirred at room temperature for 24 h. The LDH–PdTCPP complex was then washed with ethanol twice and centrifuged at 13 000 rpm for 15 min. The degree of PdTCPP loading in LDHs was determined by measuring the UV–vis absorbance at 400 nm (Soret band of PdTCPP) as the LDH matrix was dissolved with methanol; yielding at a maximal loading of 6.66 weight% of PdTCPP with respect to the LDHs.

### 2.6. Synthesis of surface functionalized LDH–FITC

FITC immobilization was performed by anchoring an amine linker on the LDH surface. This can be effectively performed using toluene as the reaction solvent as reported previously [46]. Surface conjugation of the LDH was achieved at higher temperature and the method can be described as follows: as-synthesized LDH nanoparticles (200 mg) were re-suspended in 30 mL of toluene and stirred vigorously, and then 1 mL of pure (3-aminopropyl) trimethoxysilane (APTS) was added to the reaction mixture and stirred at 90 °C for 24 h under a nitrogen purge. Finally, the nanoparticles were collected and washed repetitively with acetone and ethanol to remove the unconjugated APTS. Dry methanol was used to dissolve the FITC for successful conjugation on the LDH-NH_2_ surfaces. For this reaction, 10 mg of FITC was dissolved in the 10 mL of dry methanol, and 100 mg of the LDH-NH_2_ nanoparticles prepared above were re-suspended and stirred at room temperature for 48 h in the dark.

### 2.7. Detection of singlet oxygen generation

Generation of singlet oxygen was determined by using the 9, 10-anthracenediyl-bis(methylene) dimalonic acid (ABMDMA) probe. ABMDMA is an anthracene derivative that can be reacted with singlet oxygen to produce the corresponding endoperoxide [47]. The reaction was measured by recording the absorbance decrease in 400 nm. For the experiment, 0.1 mM of ABMDMA (in water) was mixed with LDHs-PdTCPP solids for irradiation by a diode laser at 532 nm. For the control experiment, 0.1 mM of the ABMDMA was mixed with LDHs in water for further irradiation.

### 2.8. Microscopic imaging for cellular uptake studies

The microscopic imaging of cellular uptake for LDHs was performed by using B16F10 cells at a density of 5 × 10^6^ cells/well in the 6-well plates for 24 h culture. LDH–FITC nanocomposites were added (10 *μ*g mL^−1^) and further incubated for 24 h. Cells were fixed with 3.7% (v/v) of formaldehyde and then cell membranes were disrupted by using 0.1% of Triton X-100 for 5 min. Later, 3% bovine serum albumin was added and incubated for 30 min; cells were then washed twice with PBS buffer. Rhodamine phalloidin was added and incubated at room temperature for 20 min for staining the filamentous actin skeleton. In addition, the nucleus was further stained with DAPI at a concentration of 2 mg mL^−1^ in double-distilled water and incubated for 5 min. The samples were observed under a confocal microscopic imaging system.

### 2.9. Photodynamic efficacy measurements

In the PDT experiments LDH–PdTCPP nanocomposites were irradiated with a 532 nm diode laser at a power density of 250 ± 5 mW cm^−2^. After irradiation the cells were further incubated in the culture medium for 48 h.

### 2.10. Cell viability assay

Cellular viability was determined by one of the known methods using MTT (3-(4, 5-dimethylthiazol-2-yl)-2,5-diphenyltetrazolium bromide) reagent. The photodynamic effects in B16F10 cells were observed, comparing with and without drug treatments as reported previously [48]. Cells were seeded at a density of 1 × 10^4^ cells/well in a 96-well plate and incubated at 37 °C with 5% CO_2_ for 24 h. After cell attachments, various concentrations (from 0 to 100 *μ*g mL^−1^) of the LDH samples were added and incubated for another 24 h. Then, 50 *μ*L of MTT solution (1 mg mL^−1^ of PBS solution) was added and incubated for an additional 4 h. Eventually, the whole medium was removed and the formation of violet crystals (formazan) was dissolved by DMSO for further measurements using an ELISA reader at 570 nm. The viability percentage was calculated from the absorbance. Cells devoid of treatment were taken as the control and the viability was set as 100%. The final data were expressed as a percentage of the control (mean ±standard deviation) and calculated; % viability = (mean absorbance of treated cells/mean absorbance of control) × 100.

### 2.11. Lactate dehydrogenase assay

A non-homogenous colorimetric assay was performed according to the manufacturer’s instructions for the quantification of lactate dehydrogenase levels to determine cellular membrane damages. Cells were seeded at a density of 1 × 10^5^ cells/well in a 12-well plate and incubated at 37 °C with 5% CO_2_ for 24 h. Additionally, cells were treated with various LDH samples and incubated for another 24 h. The light was irradiated for a specified time period in each respective sample-treated well and the procedure was followed according to the manufacturer’s instructions.

### 2.12. Detection of intracellular ROS

Intracellular ROS levels were determined using a dichlorodihydrofluorescein diacetate (DCFH-DA) assay. B16F10 cells at a density of 1 × 10^4^ cells/well were seeded in a 96-well plate, followed by incubation with LDH–PdTCPP nanocomposites for 24 h. For determination of the cellular ROS, cells were incubated with 20 *μ*M of H_2_DCFDA at 37 °C for 30 min. This is because DCF-DA is a hydrophobic cellular permeable probe, which can further deacetylate to DCF by the catalysis of cellular esterases. DCF is further oxidized by cellular ROS to produce 530 nm fluorescence under 485 nm excitation. The cells were scrapped and centrifuged, and fluorescent intensity of the ROS was recorded approximately 90 min after irradiation.

### 2.13. Comet assay

The DNA fragmentation was performed using a Trevigen’s Comet Assay^®^ kit. B16F10 cells were seeded at a density of 1 × 10^5^ cells/mL in 6 cm dishes. After 24 h of incubation for cell attachments, the medium was replaced with LDH–PdTCPP nanohybrid suspension in medium. After 4 h, cells were irradiated with specified light doses and further incubated for 20 h. The cells were then harvested and the DNA was extracted and mounted on slides. After mixing with low-melting agarose, gel electrophoresis was performed according to the manufacturer’s instructions. Finally, the DNA was stained with SYBR green and the tailing length was observed under a fluorescent microscope.

### 2.14. Measurement of mitochondrial membrane potential

The fluorescent probe of JC-1 is a cationic dye which exists as a monomer with green fluorescence at low membrane potential. However, the hyperpolarization can further cause the JC-1 to form red fluorescent aggregates [49]. B16F10 cells (1 × 10^4^ cells/well) were seeded in a 96-well plate, followed by incubation with LDH–PdTCPP nanohybrids for 4 h. Furthermore, the treated cells were exposed to laser irradiation for 5 min and incubated for another 24 h. Cells were then re-suspended in 500 *μ*L of PBS containing 2 mM of JC-1 and incubated at 37 °C for 30 min. The j-aggregates from the change of mitochondrial membrane potential were observed under a fluorescence microscope.

### 2.15. Animal xenograft tumour model for PDT

An *in vivo* xenograft tumour model was executed following the ‘Guide for the Care and Use of Laboratory Animals’ by the National Dong Hwa University (Hualien, Taiwan). ICR male mice (eight weeks old) weighing around 40–45 grams were purchased from LASCO (Charles River Technology, Taipei, Taiwan). Animals were handled in accordance with government animal testing guidelines. Animals were housed in a temperature (21 ± 1 °C) and humidity (55 ± 10%) controlled room within a light (12 h)/dark (12 h) cycle and were provided food and water ad libitum. Every mouse used for the study was subcutaneously injected with 200 *μ*L volume containing 1 × 10^7^ B16F10 cells to induce a solid tumour on its back and the treatment was started after the tumour volume reached an average volume of about 1200 mm^3^. The mice were randomly divided into different groups (each group with 4 animals) based on treatment conditions: two groups received an injection of normal saline as a control and the other two received LDH–PdTCPP at the tumour site (12.5 mg/kg/dose for 3 doses), each dose once for two days and then doubled (25 mg/kg/dose for more 4 doses). After treatment, one group each of control and treatment was irradiated by the 532 nm laser in the presence of the photosensitizer. Subsequently, the body weight of the animals was monitored at a regular interval and the data were analyzed. The mice were then sacrificed and the tumours were excised and weighed. Major organs such as the liver and kidney of all the groups were subjected to relative organ weight analysis. Carbon dioxide inhalation was used for euthanasia.

## 3. Results and discussion

### 3.1. Material characterization

**Figure 2.**
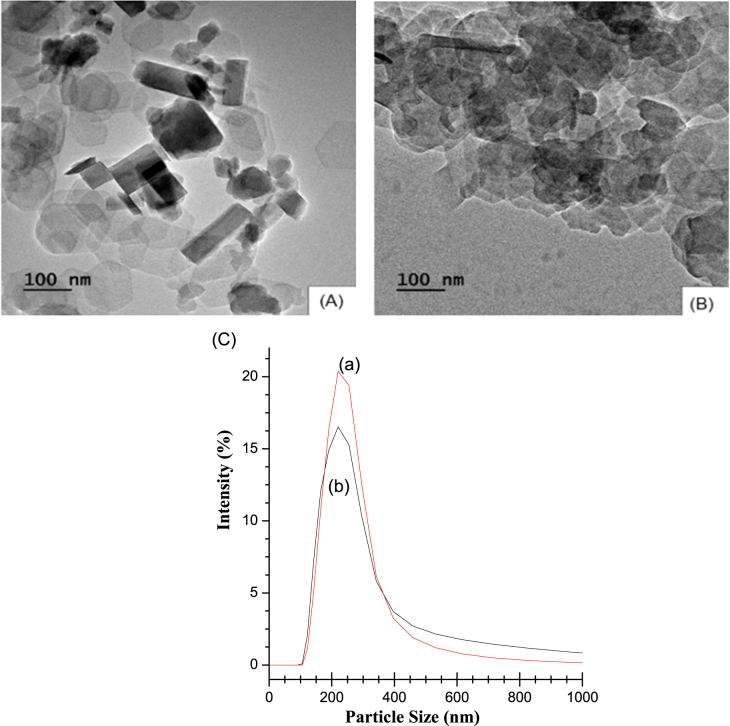
Characterization of LDH and LDH–PdTCPP for (A) TEM image of LDH, (B) TEM image of LDH–PdTCPP, (C) particle size distributions of (a) LDH–PdTCPP and (b) pristine LDH nanocomposites.

The morphology of LDH and LDH–PdTCPP was examined by TEM and the results are shown in figures 2(A) and (B). The LDH has the shape of hexagonal plates, while the LDH–PdTCPP is shaped like round plates with edges not as sharp as those of the LDH. The round shape of PdTCPP-loaded LDH is consistent with what has been reported previously [50]. The size distribution of LDH and LDH–PdTCPP was measured by dynamic light scattering (DLS) and the results are shown in figure 2(C). The average particle sizes of the pristine LDH and LDH–PdTCPP were about 212 ± 5 and 225 ± 8 nm respectively. The zeta potential value of the pristine LDH was around 22.3 ± 0.2 mV and the zeta potential value of the photosensitizer-loaded LDH was further decreased to nearly neutral (0.2 ± 0.1 mV) (table S1). The LDH frameworks have positive surface charges which highly favour cellular uptake efficiency to increase the efficiency for PDT. These results also provided indirect evidence for the incorporation of the photosensitizer in LDH. The loading of the PdTCPP into the LDH interlayers resulted in charge neutralization; therefore, an increase of aggregated LDH nanoparticles in the solution was detected by DLS measurements. The results obtained correlate to the previous findings by Choy and colleagues that LDH particle size around 200 nm has more uptake via clathrin-mediated endocytosis, whereas the larger particle size counterparts entered the cells without any specific cellular entry pathway [43].

We have analyzed the crystal structure of LDH during incorporation of PdTCPP molecules by PXRD (figure S1). Pristine LDH has shown the typical diffraction pattern of hydrotalcite with hexagonal crystal lattice in accordance with the previous reports [51]. The d(003) peak at 11.66° giving a d-spacing of 0.76 nm was typical for Mg_2_Al-LDH with chloride as the counterion. The existence of a diffraction peak at d(110) and d(113) in the LDH structure indicated the LDH with hydroxide layer as a two-dimensional crystal with a typical hexagonal arrangement of layers. The shift of the d(003) peak to a lower angle revealed a slight expansion of the interlayer distance due to the intercalation of the PdTCPP molecules in the interlayer. Apparently, PdTCPP is arranged in a horizontal arrangement in the interlayer spacing.

**Figure 3.**
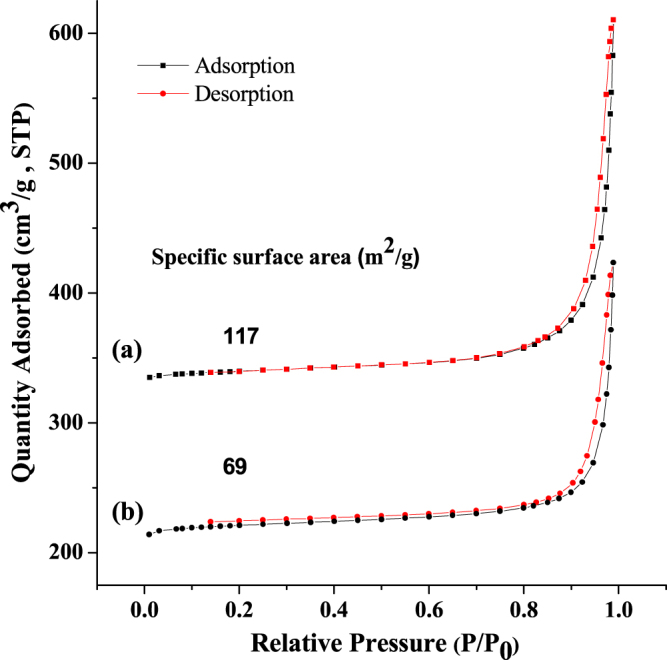
Nitrogen adsorption–desorption isotherms of (a) LDH and (b) LDH–PdTCPP nanocomposites.

Figure 3 shows the nitrogen adsorption–desorption isotherms from analysis using the BET method. Pristine LDH nanoparticles have a specific surface area of 117 m^2^ g^−1^ (figure 3(a)), whereas PdTCPP-loaded LDH resulted in a significant decrease in specific surface area from 117 m^2^ g^−1^ to 69 m^2^ g^−1^ (figure 3(b) and table S1). The drastic decrease in specific surface area for the PdTCPP-intercalated sample may be attributed to the change in the textured porosity as a result of strong interactions between the PdTCPP molecules and LDH matrix. Furthermore, pristine LDH nanoparticles showed a pore volume of 0.44 cm^3^ g^−1^ while the intercalation of PdTCPP resulted in a large decrease of pore volume to 0.34 cm^3^ g^−1^ (table S1), which indicated that the PdTCPP molecules were intercalated in the gallery of LDH interlayers.

**Figure 4.**
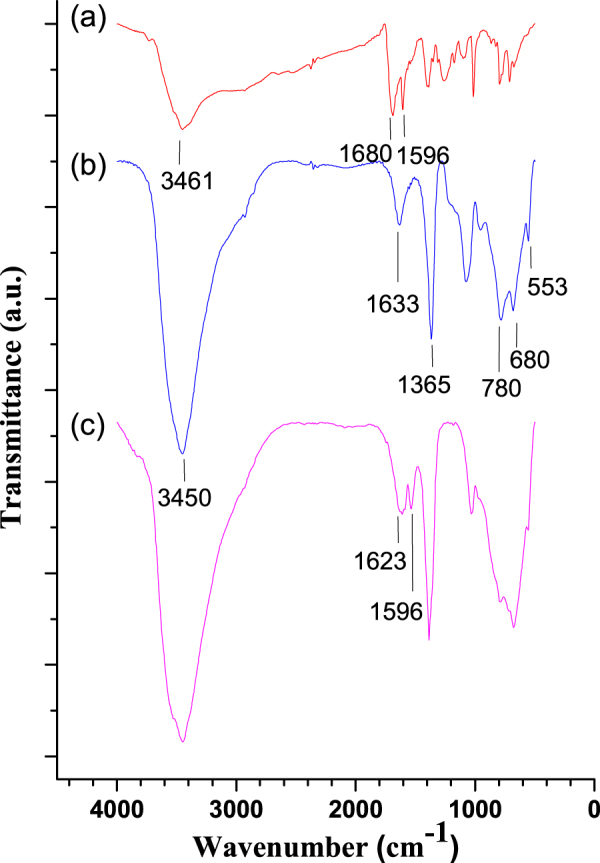
FT-IR spectra of (a) pure PdTCPP, (b) pristine MgAl–LDH and (c) LDH–PdTCPP nanocomposites.

The incorporation of PdTCPP molecules in the LDH layers was confirmed by FT-IR spectra (figure 4). The infrared spectrum of PdTCPP molecules (figure 4(a)) showed characteristic bands of PdTCPP molecules of ν(OH) at 3461 cm^−1^; the peaks at 1680 and 1596 cm^−1^ were attributed to the stretching vibration of ν(C=O) and aromatic ring, respectively. The framework of pristine-LDH (figure 4(b)) was confirmed by a broad band of ν(O-H) at around 3450 cm^−1^ and a weak peak at 1633 cm^−1^ (H_2_O deformation) due to the intercalation of water in the LDH matrix. Bands at 553 and 680 cm^−1^ are attributed to the lattice vibration of M–O and M–O–M (M = Mg, Al) respectively, confirming the formation of frameworks. The peak at 1365 cm^−1^ was attributed to the stretching vibration of 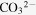 converted from atmospheric CO_2_ captured during synthetic processes [52, 53]. The typical IR modes of the PdTCPP backbone such as the stretching vibration of the aromatic ring at 1596 cm^−1^ and ν(O-H) at 1623 cm^−1^ in the LDH–PdTCPP nanohybrids were observed (figure 4(c)), confirming the successful loading of PdTCPP in the LDH.

To evaluate the delivery efficacy of LDH–PdTCPP nanocomposites, it is important to determine the loading amounts of PdTCPP molecules in the LDH nanocarrier. Thermogravimetric analysis (TGA) can be used to measure the weight loss percentages of solid samples as a function of temperature under a nitrogen atmosphere (figure S2). The main weight loss of the PdTCPP solids was clearly observed in the region of 403–525 °C, which was attributed to decomposition of PdTCPP molecules, corresponding to a strong peak at 403 °C for about 17% weight loss (figure S2(a)). For the pristine LDH sample (figure S2(b)), a weight loss was observed at around 343 °C, which was related to the decomposition of the LDH matrix through the removal of interlayer anions and further dehydroxylation of the layers. Figure S2(c) shows the thermal decomposition of the PdTCPP-intercalated LDH samples. To compare with the weight loss from the pristine LDH and LDH–PdTCPP samples, it shows an additional peak in the temperature range from 389 to 553 °C, which is assigned to the PdTCPP molecules loaded in the interlayers of the LDHs. This temperature is higher than the decomposition temperature of pure PdTCPP solids (about 525 °C). The broad degradation peak around 350 °C is due to PdTCPP intercalated in LDH to further confirm the enhanced thermal stability of the intercalated PdTCPP in the gallery of LDHs. The weight loss value of the LDH–PdTCPP is about 12%.

### 3.2. Absorption spectra and fluorescence emission spectra of LDH nanocomposites

**Figure 5.**
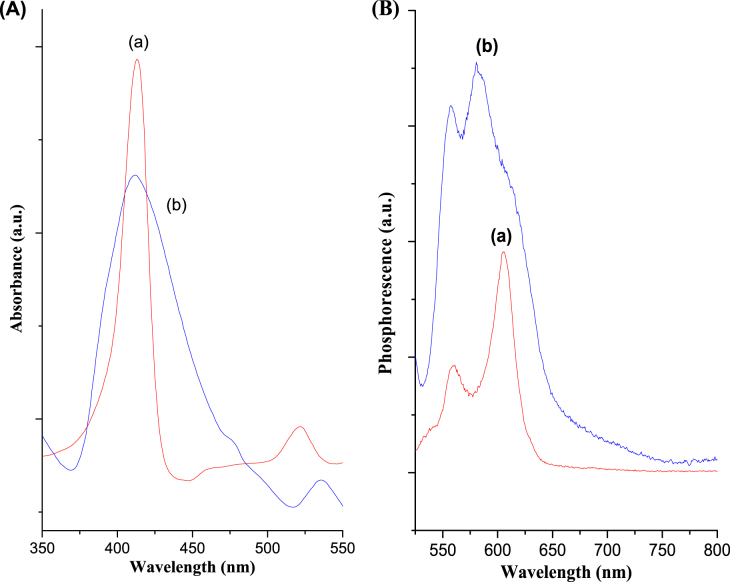
(A) UV–vis absorption spectra and (B) fluorescence emission spectra of (a) PdTCPP and (b) LDH–PdTCPP in solution.

The aggregation behaviour of porphyrins can be characterized from their UV–vis spectra, which are prone to form oligomers due to the macrocycle–macrocycle interactions [54]. This behaviour may cause fast vibrational relaxation to their ground state for further decreasing the quantum yields. The UV–vis spectra of LDH–PdTCPP nanohybrids (figure 5(A-b)) were significantly different from the spectra of the PdTCPP solutions (figure 5(A-a)). In comparison with the sharp Soret band of the spectrum in the PdTCPP solution, the intercalation of PdTCPP into LDH resulted in a broadening Soret band with a maximum absorbance at 412 nm and a Q band that has red shifted about 2 nm [55]. These results indicate the electrostatic coupling of the PdTCPP chromophores in between the layered matrices of the LDHs. The obtained results are similar to those reported for MgAl–LDH–PdTCPP previously [56]. Relative to the PdTCPP solution (fluorescent emission at 606 nm in figure 5(B-a)), the fluorescence emission spectrum of LDH–PdTCPP nanocomposites was slightly shifted to 580 nm (figure 5(B-b)) and the intensity of the emission band was also increased. These results confirm that the PdTCPP molecules retained their photoactivity in the interlayer structures and the ion-exchange processes did not damage the PdTCPP structures. Evidently, the variations in the fluorescence properties are due to the hydrogen-bonding interactions of PdTCPP molecules with the layered hydroxyl surfaces, and thus changes in the PdTCPP microenvironments.

**Figure 6.**
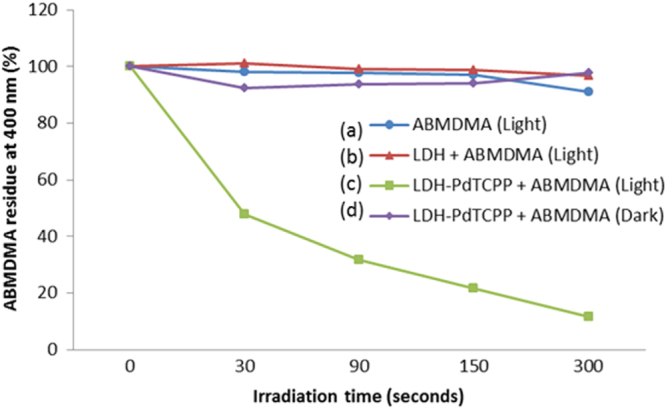
Singlet oxygen generation of (a) ABMDMA solution, (b) LDH with ABMDMA, (c) LDH–PdTCPP with ABMDMA and (d) LDH–PdTCPP with ABMDMA measured as an absorbance change at 400 nm versus irradiation time at 532 nm.

Highly reactive oxygen (singlet oxygen species) is believed to play an important role in the PDT effects especially against malignant tumours. The combination of nanoparticle drug delivery systems with different photosensitizers can generate these species in order to treat various surface diseases of the skin. We performed the singlet oxygen activity using the LDH–PdTCPP sample and further compared it with pristine LDH under light and dark conditions. The efficiency of singlet oxygen generation was determined by photodegradation of ABMDMA, which showed a decrease of absorbance at 400 nm in the presence of the singlet oxygen species. Thus, we measured the decrease of absorbance at 400 nm in the presence of various samples as a function of exposure time under irradiation with a 532 nm laser as presented in figure 6. The LDH–PdTCPP sample (figure 6(c)) showed fast loss of ABMDMA absorbance to compare with pristine LDH (figure 6(b)) which indicated that the generation of singlet oxygen was very efficient. In particular, the 50 s of laser irradiation caused about 60% absorbent reduction for the LDH–PdTCPP sample, which then declined rapidly after 300 s of laser irradiation. For the control experiments, the laser irradiation of the ABMDMA solution (figure 6(a)) and pristine LDH (figure 6(b)) showed an insignificant decrease in absorbance at 400 nm and the LDH–PdTCPP sample without laser irradiation also showed no singlet oxygen activity. These results suggested that LDH–PdTCPP nanocomposites have a great photodynamic effect, which can only generate singlet oxygen under laser irradiation. Figure S3 shows the photostability of LDH–PdTCPP nanocomposites in comparison with the pristine PdTCPP solution. After continuous irradiation with a 532 nm laser for 35 min, the absorbance of the free PdTCPP solution decreased about 85% (figure S3(a)), while only 10% loss was observed for the LDH–PdTCPP nanocomposites respectively (figure S3(b)). The results indicated that the use of LDH as the nanocarrier to intercalate PdTCPP molecules into the gallery of LDH can efficiently protect the photosensitizer molecules against the photobleaching effect, and that the LDH–PdTCPP possesses a better resistance than the pristine PdTCPP solution. Thus, the studies clarified that the LDH nanoformulation carrying the PdTCPP photosensitizer has high stability and efficiency for the development of PDT. The use of LDH nanoparticles as the delivery vehicle possesses the characteristics of biodegradability and biocompatibility, which can release photosensitizer in the weak acidic pH of tumour tissues and further degrade and metabolize the LDH frameworks.

### 3.3. Cellular uptake studies

**Figure 7.**
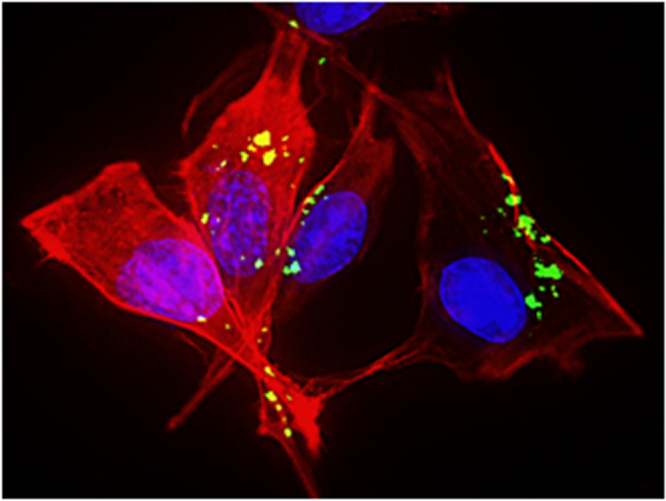
Fluorescence images of B16F10 cells after incubation with LDH–FITC nanocomposites (green: FITC–LDHs, red: cytoskeleton, blue: nucleus).

The internalization of LDH nanoparticles into the B16F10 cells was observed directly using fluorescence microscopy. We conjugated FITC to LDH nanoparticles using the amine-modified LDH surfaces and intracellular fluorescence was observed using fluorescence microscopy (figure 7). After incubation of FITC-labelled LDH nanocomposites for 24 h, we observed the blue DAPI fluorescence from the stained nucleus, the red fluorescence from the cytoskeleton and green fluorescence from the FITC emission of the LDH nanocomposites (figure 7). Furthermore, the localization of LDH–FITC nanocomposites in the cell was observed, which indicates the successful endocytosis of the nanoparticles and is in agreement with the previous reports by Choy *et al* [57] and Xu *et al* [58] observed in NIH3T3 cells.

### 3.4. Phototoxic studies of the LDH–PdTCPP nanocomposites

**Figure 8.**
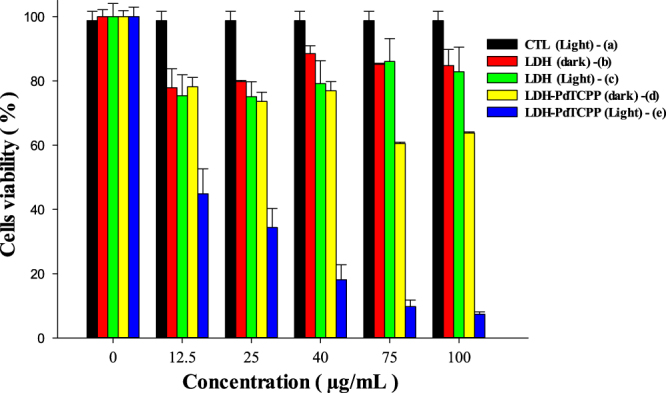
The PDT performance of (a) control cells under irradiation, (b) pristine LDH in dark conditions, (c) pristine LDH under irradiation, (d) LDH–PdTCPP nanohybrids in dark conditions and (e) LDH–PdTCPP nanohybrids under irradiation.

Cell viability was evaluated using the MTT (3-[4, 5-dimethylthiazol-2-yl]-2, 5-diphenyltetrazolium bromide) assay either with or without laser irradiation with different concentrations of pristine LDH and LDH–PdTCPP nanocomposites. The pristine LDH nanoparticles showed only a slight decrease in cell viability within the tested concentration range even as high as 100 *μ*g mL^−1^ in irradiation (figure 8(b)) or no-irradiation (figure 8(c)) conditions, while a higher concentration of LDH–PdTCPP nanocomposites (100 *μ*g mL^−1^) had a further decrease of cell viability to 65% in dark conditions (figure 8(d)), which may come from the side effects of leaching metals. However, the LDH–PdTCPP-treated group showed high efficiency of PDT, and therefore the cell viability was dramatically decreased under laser irradiation in a concentration-dependent manner (figure 8(e)).

The possibility of necrosis in cell cytotoxicity was examined by lactate dehydrogenase assay. A key signature of necrotic cell death is the permeabilization of the plasma membrane. This episode can be quantified by measuring the release of the intracellular lactate dehydrogenase enzyme [59]. For this experiment, the maximal release (positive control) was obtained by the treatment of cells (no nanoparticles) with 0.5% triton X-100 for 10 min at room temperature. Compared with the dark control experiment (no nanoparticle treatment), laser-irradiated cells showed an increase in lactate dehydrogenase release (figure S4(c)) which was in accordance to the previous findings that infrared light can cause a very mild lactate dehydrogenase release [60]. However, the pristine LDH (figure S4(d)) and LDH–PdTCPP nanocomposites (figure S4(e)) did not show any substantial increase in the release of lactate dehydrogenase after treatment with a concentration (25 *μ*g mL^−1^) for 24 h either with (figure S4(f)) or without irradiation (figure S4(e)). The results showed that both the pristine LDH and the LDH–PdTCPP nanocomposites are highly biocompatible because the nanoparticle-treated cells can retain their membrane integrity to further decrease the side effects from the unexpected necrotic cell death.

**Figure 9.**
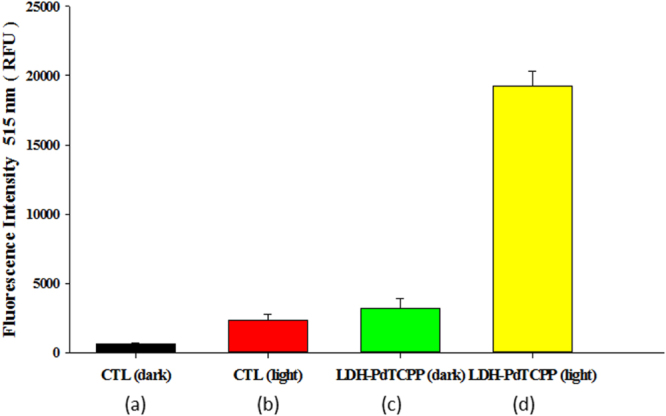
Determination of the ROS levels in B16F10 cells using DCFH-DA of (a) dark conditions without nanoparticles, (b) laser irradiation without nanoparticles, (c) dark conditions with LDH–PdTCPP nanocomposites and (d) laser irradiation with LDH–PdTCPP nanocomposites. DCF fluorescence was detected by ELISA reader.

The nanocomposites were capable of increasing the intracellular ROS level through light activation, which was detected by using the H_2_DCFDA fluorescent probe. The use of PDT to generate the intracellular ROS can further oxidize DCFH to fluorescent DCF. In comparison to the control groups in the dark (figure 9(a)) and light (figure 9(b)), the treatment of LDH–PdTCPP nanocomposites induced significant levels of ROS under laser irradiation (figure 9(d)). The ROS intensity induced by LDH–PdTCPP nanocomposites without laser irradiation (figure 9(c)) was remarkably lower than the ROS generated in the presence of laser irradiation. From the experiments of the PDT effects and singlet oxygen production, we confirmed the LDH–PdTCPP nanocomposites have the potential to develop PDT applications in comprehensive cancer treatment.

**Figure 10.**
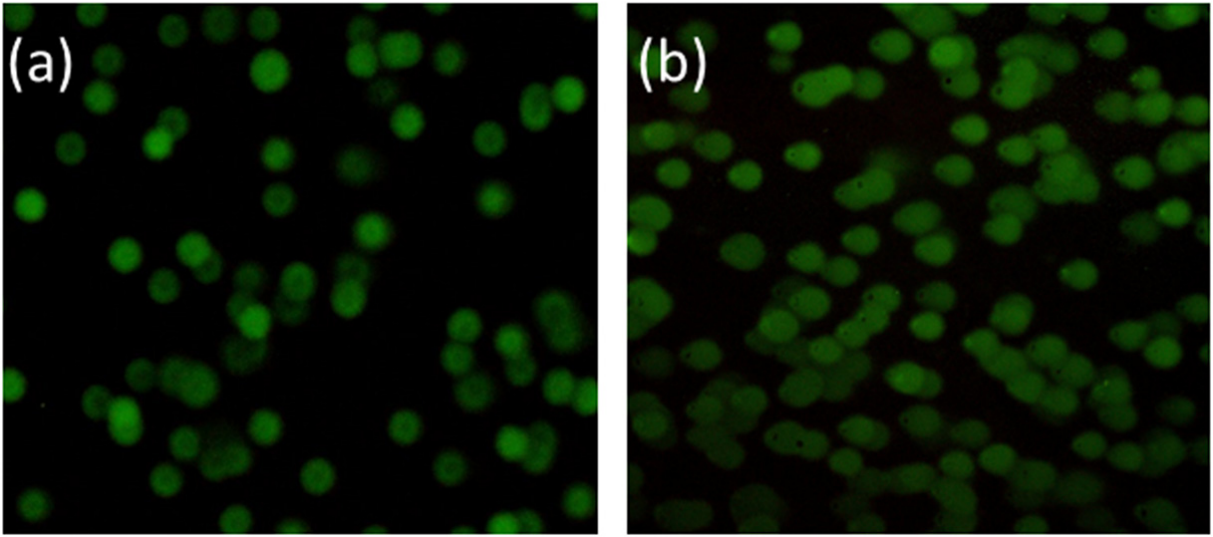
DNA fragmentation (comet assay), (a) control without laser irradiation (undamaged cells) and (b) cells treated with LDH–PdTCPP nanocomposites at 25 *μ*g mL^−1^ under laser irradiation. Nuclei were stained with SYBR^®^ green dye.

Recent studies have suggested that PDT can result in base oxidation, cross-linking of DNA strands, heat shock of proteins and sister chromatid exchange [61]. Our studies showed that the LDH–PdTCPP nanocomposites involved in the generation of intracellular ROS of singlet oxygen have a very short life span of less than 1 s. The generated ROS species need to be very close to a DNA strand to cause oxidative damages. In addition, PDT is capable of damaging cytoplasmic proteins and mitochondria activity leading to the arrest of cell cycles and resulting in cell apoptosis [62]. Our results showed that the use of LDH–PdTCPP nanocomposites for PDT can cause the DNA damages through oxidative breaks, which were in good agreement with the previous reports of Miller *et al* confirming the PDT effects [62]. The damage level of DNA was evaluated by the tail length, which was a bit more in the cells treated with the LDH–PdTCPP nanocomposites followed by laser irradiation (figure 10(b)) than in the untreated control groups (figure 10(a)).

**Figure 11.**
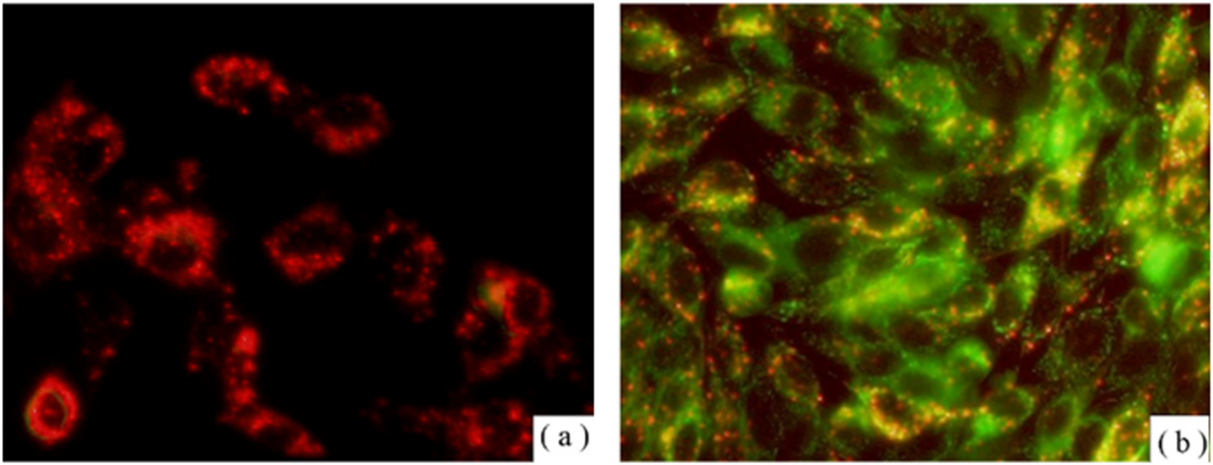
Measurement of mitochondrial membrane potential of LDH–PdTCPP-treated B16F10 cells (a) without laser irradiation (J-aggregation of red fluorescence) and (b) with laser irradiation (J-monomer of green fluorescence).

Figure 11 shows the PDT effects for the oxidative damages in mitochondria under the treatment of LDH–PdTCPP nanocomposites. In the dark environment, the treated cells did not detect the loss of mitochondrial membrane potential, and thus the JC-1 dye showed the aggregate type with red fluorescence (figure 11(a)). For the laser irradiation, the LDH–PdTCPP-treated cells showed loss of mitochondrial membrane potential of membrane potential disruption. Therefore, the JC-1 dye is predominantly a monomer with green fluorescence (figure 11(b)). The heavy dissipation of the mitochondrial membrane potential may cause the decline of mitochondria ATP production, thereby causing mitochondria dysfunction leading to irreversible cell death.

### 3.5. *In vivo* photodynamic efficacy

**Figure 12.**
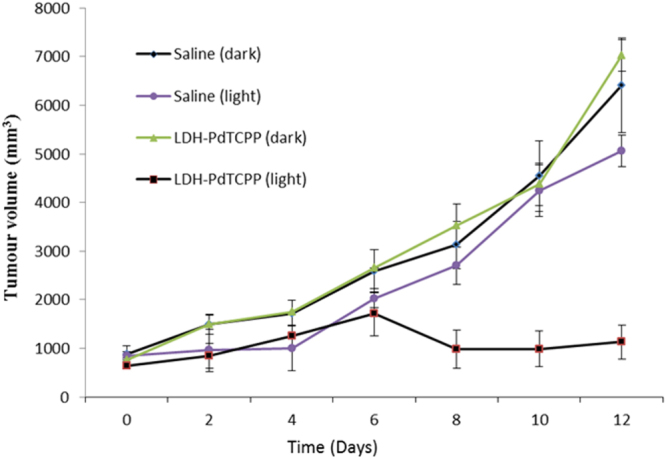
Volume analysis of B16F10 tumour as a function of time after implantation in mice treated with LDH–PdTCPP at specified doses and irradiated with 532 nm, another group with normal saline (control). Each group (*n* = 4) was subjected to the 12 day course of treatment.

Antitumour efficacy was evaluated using a xenograft model in mice. After a subcutaneous injection of the B16F10 cell line, we started to treat with LDH–PdTCPP formulation with an optimized dose (see experimental section). The injected LDH–PdTCPP nanocomposites significantly reduced the tumour volume (by sevenfold; see figure 12) compared to the control (saline) mice group. This study revealed that the light alone and the nanocarrier systems (LDH–PdTCPP) in the dark have no effect. Similarly, photosensitizer alone was administered at the same dose as to the other group, which resulted in an undesirable precipitation in saline (the solubility of the PdTCPP solids is indeed poor). Besides, we used DMSO as co-solvent to solubilize the drug, which led to the demise of the animals due to adverse effects of PdTCPP and DMSO toxicity. Previously, our group has reported that the visible light irradiated alone for untreated cells at a wavelength of 532 nm has no significant effect on melanoma cell line [30]. It is evident the *in vivo* tumour volume reduction is induced by apoptosis with the delivery of photosensitizer upon light irradiation. We also observed supportive evidence obtained *in vitro* such as DNA fragmentation by the comet assay (figure 10) and mitochondrial membrane potential by the JC-1 assay (figure 11). Thus, we demonstrated a higher inhibition of tumour growth with the photosensitizer-loaded biocompatible nanovehicle. The simultaneous record of body weight change (figure S5) in mice means that the significant reduction in tumour growth with no body weight changes signifies that the nanoformulation was biocompatible.

Table 1 illustrates the relative liver and kidney organ weights of mice in each group administered with normal saline (control) and LDH–PdTCPP nanocomposites under light irradiation. There is no significant difference in the relative liver and kidney weights of either the control or treatment groups (LDH–PdTCPP) consecutively for 12 days. These results in addition to animal body weight change showed that the formulation was highly biocompatible. All animals remained in healthy condition throughout the experimental period with no further complications. Herewith, the nano-photosensitizer formulation reduced the tumour, in both *in vitro* and *in vivo* experiments.

**Table 1.** Relative organ (liver and kidney) weight analysis of LDH–PdTCPP treated mice for 12 days with a tumour.

	Relative organ weight (g/100 g of body weight)
Group	Liver	Kidney
Control	7.4 ± 0.9	2.6 ± 0.6
LDH–PdTCPP (Light)	7.8 ± 0.1	2.7 ± 0.3

## 4. Conclusions

We reported the intercalation of the palladium porphyrin photosensitizer (PdTCPP) in the gallery of LDH nanoparticles for photodynamic therapy to induce cytotoxicity against B16F10 melanoma cancer cell lines. The use of LDH as a delivery vehicle has the advantages of facilitated nanoparticle internalization into the cellular cytoplasm and further increased intracellular density of singlet oxygen upon photo-irradiation. The large surface areas of LDH nanoparticles with biodegradable and biocompatible properties can provide solubility enhancement to the hydrophobic PdTCPP photosensitizer. The *in vivo* studies also demonstrated high efficiency for PDT performance to reduce the tumour growth by sevenfold compared to the control mice group. The LDH–PdTCPP nanocomposites also showed low cytotoxicity, with no significant change in body weight of mice and variation of relative organ weight to demonstrate that the nanocomposites were biosafe and could be a promising drug delivery vehicle for PDT. The incorporation of PdTCPP in LDH nanoparticles can also serve as an *in vivo* contrast agent for oxygen sensing to image tumour hypoxia, which has great promise as a useful nano-platform for cancer theranostics.
